# Quality of Life in Adults with Tetralogy of Fallot: Physical Limitations and Psychological Well-Being

**DOI:** 10.3390/jcdd12050178

**Published:** 2025-05-07

**Authors:** Panagiotis Zachos, Evelina Pappa, Nikias Milaras, Vasileios Nevras, Paschalis Karakasis, Nikolaos Ktenopoulos, Maria Karakosta, Alkistis-Eleni Kalesi, Nearchos Kasinos, Anastasios Theodosis Georgilas, Stefanos Despotopoulos, Sotiria Apostolopoulou, Dimitrios Niakas

**Affiliations:** 1 Department of Health Economics, School of Medicine, National and Kapodistrian University of Athens, 10679 Athens, Greece; p.zahos@yahoo.gr (P.Z.); dimitris.niakas@gmail.com (D.N.); 2 Pediatric Cardiology and Adult Congenital Heart Disease Department, General Hospital of Karditsa, Tavropou (Terma), 43100 Karditsa, Greece; 3 Faculty of Social Sciences, Hellenic Open University, Riga Fereou 169 & Tsamadou, 26222 Patras, Greece; evanpappa@gmail.com; 4 Medical School, National and Kapodistrian University of Athens, Vasilissis Sofias 114, 10679 Athens, Greece; pakar15@hotmail.com (P.K.); nikosktenop@gmail.com (N.K.); 5 Cardiology Department, General Hospital of Karditsa, Tavropou (Terma), 43100 Karditsa, Greece; nevras_bill@hotmail.gr; 6 Echocardiography Training Center “D. Beldekos”, Cardiology Department, Tzaneio General Hospital of Pireaus, 18536 Attica, Greece; mariakarakosta@hotmail.com (M.K.); eileen_calessis@yahoo.gr (A.-E.K.); nearchoskassinos@gmail.com (N.K.); medtheodosis@hotmail.com (A.T.G.); 7 Pediatric Cardiology and Adult Congenital Heart Disease Department, Onassis Cardiac Surgery Center, 17674 Kallithea, Greece; stefanosdespotopoulos@gmail.com (S.D.); sotiria.apostolopoulou@gmail.com (S.A.)

**Keywords:** tetralogy of Fallot, health-related quality of life, SF-36, EQ-5D, adult congenital heart disease, physical functioning, mental health

## Abstract

**Background:** Advances in medical care of patients with tetralogy of Fallot (ToF) have significantly altered the natural course of the disease by prolonging life. Thus, our focus has now shifted to exploring the health-related quality of life (HRQoL) of those patients. This study sought to explore the HRQoL of adult patients operated on for ToF using two validated instruments—the SF-36 and EQ-5D—that highlight both physical and mental aspects of the disease. **Methods:** A total of 115 individuals (53 ToF patients and 60 healthy controls) were recruited for the purposes of this study. HRQoL was assessed through the SF-36 and EQ-5D instruments. Comparisons were made between ToF patients and controls with subgroup analyses based on sex and age. **Results:** ToF patients reported significantly poorer HRQoL in the physical domains, namely Physical Functioning, Role Physical, General Health, and Physical Component Summary of the SF-36 when compared to controls (*p* < 0.05). Interestingly, there was a trend towards lower Bodily Pain and better Vitality scores in ToF subjects. Age influenced HRQoL, with older respondents rating their physical health lower than younger patients and controls (*p* < 0.05). EQ-5D VAS scores indicated that ToF patients perceived their overall health worse than controls (80.02 vs. 86.92, *p* < 0.001), with Anxiety/Depression being the most frequently reported problem (45.3%). Controls reported better HRQoL than ToF patients across all SF-36 domains in both health states (EQ-5D = 1 and EQ-5D > 1), except for Bodily Pain and Vitality in EQ-5D = 1. Significant differences were observed in Physical Functioning, Role Physical, General Health, Vitality, and Physical Component Score. Notably, ToF patients with EQ-5D = 1 showed unexpectedly higher Vitality scores than controls; however, this advantage diminished significantly in the EQ-5D > 1 group. **Conclusions:** Adult ToF survivors experience significant physical limitations as expected, while mental health seems to remain relatively unaffected compared to healthy peers. These findings underscore the importance of HRQoL assessment in patients with congenital heart disease and the need for disease-specific HRQoL instruments.

## 1. Introduction

With significant advancements in medical and surgical management of complex congenital heart disease (CHD), such as tetralogy of Fallot (ToF), the focus has shifted from hard endpoints like mortality to exploring the quality of life (QoL) in these patients. ToF, recognized as the most prevalent complex cyanotic CHD (affecting 3–6 per 10,000 live births and accounting for 10% of adult CHD), is more commonly observed in its surgically repaired form among adults [1,2].

The etiology of ToF remains unclear, though genetic studies suggest a potential link to gene polymorphisms [3]. Diagnosis is typically confirmed via echocardiography, prompting early surgical intervention. While surgery is not curative, approximately 50% of patients require reoperations during their lifetime, significantly impacting their QoL [4].

The advent of neonatal repair techniques has enabled around 90% of patients with complex cardiac lesions to survive into adulthood, forming a growing population of adults with congenital heart disease [5]. However, extended survival does not always equate to better health or health-related quality of life (HRQoL), which, as defined by the CDC, encompasses an individual’s perceived physical and mental health over time. Studies assessing HRQoL in surgically repaired ToF patients using tools such as SF-36, KINDL-R, and EQ-5D revealed that ToF patients often score lower than healthy, age-matched controls in perceived health metrics [6]. Interestingly, some ToF patients report health and mental well-being comparable to or better than healthy individuals, explained by phenomena such as the disability paradox (patients with health impairments reporting unexpectedly higher HRQoL as an adaptive mechanism to illness), response shift (a change in internal standards as an adaptive mechanism to illness), and sense of coherence (a psychological resilience trait wherein patients perceive their lives as more manageable and meaningful as an adaptive mechanism to illness) [7,8,9].

The purpose of this study was to evaluate the HRQoL of adult ToF patients as assessed using the SF-36 and EQ-5D-3L questionnaires and directly compare it to a control group of age- and sex-matched individuals.

## 2. Patients and Methods

This was a prospective study conducted in two hospitals in Greece from 1 January 2017 to 1 January 2024. Patients were identified through a review of clinic schedules with a documented primary diagnosis of ToF and approached for enrollment in the study. Adult patients were included (>18 y) who were able to correctly complete the SF-36 and EQ-5D-3L questionnaires. For the purposes of this study, 53 adult patients with ToF were recruited, as well as 60 sex- and age-matched healthy counterparts. The control group was selected during the pandemic, in the COVID-19 vaccination offices of the two hospitals, where adult subjects with no known comorbidities were asked to participate.

Health-related quality of life was assessed by using two generic instruments, the SF-36 Health Survey and the EQ-5D. Both instruments have been used widely, alone or in parallel, in health studies for measuring self-assessed HRQoL; however, they have different structures. The first measures health across a number of dimensions (scales) interpreted separately, whereas the second is classified as a multiattribute health system from which a single index is computed. The latter is based on utility theory and is exploited in cost–utility analyses.

The SF-36 Health Survey is a widely used questionnaire for assessing HRQoL [10]. It is designed to evaluate overall health status across multiple dimensions and is commonly used in clinical practice, research, and population health studies. It consists of 36 questions that measure eight dimensions of health. Each dimension is scored on a 0–100 scale, with higher scores indicating better health or functioning. Results can also be summarized into two components, namely the Physical Component Summary and the Mental Component Summary. The SF-36 is a non-disease-specific questionnaire and has been validated in the general Greek population [11].

The EQ-5D is another standardized instrument that is designed to measure HRQoL. It is a simple form that measures health across five dimensions [12]. Each dimension is assessed at one of three levels of severity (e.g., no problems, some problems, extreme problems). Each combination of responses across the five dimensions represents a unique health state. For example, a health state described as 12,345 corresponds to different levels of severity for each dimension. Alongside the dimensions, the EQ-5D includes a Visual Analogue Scale (VAS), where respondents rate their overall health on a scale from 0 to 100. Zero corresponds to the worst imaginable health, and 100 to the best. Health states are converted into a single summary index (utility score) EQ index using a country-specific value set. Utility scores typically range from 1.0 (perfect health) to 0.0 (death). This instrument has also been validated in the Greek population [13].

## 3. Statistical Analysis

Data were assessed for normality according to the Kolmogorov–Smirnov and Shapiro–Wilk tests. Descriptive statistics were applied in order to present participants’ characteristics. The χ^2^ (chi-square) test was used to examine differences in frequencies between ToF patients and the controls. Parametric and non-parametric tests for independent samples, as well as the *t*-test/Mann–Whitney test, were used to examine differences in average means/average ranks, respectively, and ANOVA/Kruskal–Wallis tests were used for differences in average means/ranks, respectively, between groups differing in key variable of age. The statistical significance was set at *p* < 0.05. All statistical analyses were conducted using SPSS 22.0.

## 4. Results

The sample distribution is given below in Table 1. One hundred fifteen individuals, divided into 53 ToF patients and 60 healthy peers, participated in the study. The sample consisted of seventy-four (65.5%) males, with a mean age of 31.8 years. All ToF patients had complete repair performed with a transannular patch. 13 of 17 female patients and 28 of 35 male patients underwent transcatheter pulmonary valve replacement in adulthood due to insufficiency, leading to a 74% rate of reoperation in our cohort. No patient in our cohort underwent surgical pulmonary valve replacement; all reinterventions were performed via transcatheter pulmonary valve implantation.

Table 2 shows the results of assessing HRQoL based on SF-36 scales and summary scores, and on the EQ-5D index and VAS, between ToF survivors and their controls. Physical Functioning, Role Physical, General Health scales, PCS summary score, and EQ-VAS had statistically significant results. As expected, adult ToF survivors rate their health lower compared to age- and gender-matched normal respondents. After examining HRQoL differences between genders in the total sample and within the patient and control subgroups, a statistically significant difference was found only in the patient subgroup on the Role Physical scale, where females reported higher scores than males (Mann–Whitney, *p* < 0.05).

With respect to the age variable, older respondents reported physical problems and rated their health lower on Physical Functioning in the total sample and in patient/control subgroups and on Physical Functioning and PCS summary score in the control group (Kruskal–Wallis, *p* < 0.05).

Mean EQ index and VAS also presented in Table 2 have shown that health condition is statistically significant for EQ VAS scale, with adult ToF patients rating their health as worse on the VAS (80.02 vs. 86.92 for controls, *p* < 0.001). Even though differences are reflected in the assigned tariff values (0.81 vs. 0.89, for controls, *p* > 0.05), they were statistically insignificant.

Table 3 presents the percentage of individuals reporting problems in each of the EQ-5D dimensions within patient–control subgroups. In four out of five dimensions, Mobility, Self-care, Usual Activities, and Pain/Discomfort, no extreme problems were reported by the ToF patients. For them, Anxiety/Depression was the most frequently reported problem (45.3% of the sample), and self-care was the least frequently declared problem (1.9%). Pain/discomfort was reported by 22.6% of the sample. Mobility and Usual Activities were reported by 13.2% of the sample. In the control subgroup, the highest percentages of reporting a moderate or severe problem in the EQ-5D dimensions were observed in the Anxiety/Depression dimension (48.35%) and the lowest percentages (0.0%) in Mobility. Differences were observed only in two dimensions. Surprisingly, higher percentages of healthy peers reported problems in the Anxiety/Depression dimension (48.3% vs. 45.3% for ToF patients, *p* > 0.05), whereas in the Mobility dimension, ToF patients reported moderate problems and healthy peers reported having no problems.

Comparing SF-36 scale and summary scores for ToF patients and controls into two health states, i.e., reporting no problems (EQ-5D = 1) and reporting any problems (EQ-5D > 1) (Table 4), controls report better HRQoL than ToF patients in every aspect of the SF-36 questionnaire in both health states except BP and VT in health state EQ-5D = 1, achieving statistical significance for PF, RP, GH, VT and PSC. Vitality was unexpectedly higher in patients with EQ-5D = 1 than in controls, although it decreased substantially in the EQ-5D < 1 group.

In our study, patients with tetralogy of Fallot who had undergone reoperation did not exhibit a significant decline in HRQoL compared to those without reintervention. Despite the potential for additional procedures to impact physical or psychological well-being, HRQoL scores remained relatively stable across both groups. This suggests that, when appropriately managed, reoperation may not adversely affect patients’ overall perception of their health and daily functioning.

Spearman rank correlations between each of the domains of the EQ-5D and the SF-36 scales are presented in Table 5. The EQ-5D Mobility dimension is weakly correlated with SF-36 Mental Health (*p* = −0.31) and MCS score (*p* = −0.28), and unexpectedly not correlated with comparable scales such as Physical Functioning. The EQ-5D Self-care dimension is not correlated with any SF-36 scale, whereas EQ-5D Usual Activities have weak correlations with SF-36 Vitality (*p* = −0.34) and moderate correlations with SF-36 Mental Health (*p* = −0.47) and MCS (*p* = −0.46). EQ-5D Pain/Discomfort is moderately correlated with five SF-36 scales, Physical Functioning (*p* = −0.47), Bodily Pain (*p* = −0.58), Vitality (*p* = −0.54), Mental Health (*p* = −0.47), and MCS (*p* = −0.45) out of seven statistically significant correlations. EQ-5D Anxiety/Depression has mainly moderate correlations with SF-36 Vitality (*p* = −0.50), Social Functioning (*p* = −0.50), Mental Health (*p* = −0.41), and the MCS scale (*p* = −0.53). The EQ index has strong correlations with SF-36 Vitality (*p* = −0.62), Mental Health (*p* = −0.60), and MCS summary scale (*p* = −0.65), while VAS has only moderate correlations with two out of six statistically significant correlations, SF-36 vitality (*p* = −0.44), and the MCS summary scale (*p* = −0.49).

## 5. Discussion

This study provides valuable insights into the HRQoL of adult patients with ToF compared to age- and sex-matched healthy counterparts. Significant survival increase due to surgical and clinical care improvements resulted in an increased adult population—patients with ToF. Besides the multiple medical issues adult patients with ToF face, there are also psychological and social challenges that influence their quality of life. In the present study, HRQL was measured by using two widely used, reliable, and validated generic instruments (SF-36 and EQ-5D) in order to highlight the multidimensional impact of ToF on both physical and mental health. Previous studies assessed the health-related quality of life of adult patients with ToF using generic questionnaires, mainly the SF-36 [13], and only two studies used EQ-5D [14,15].

Our results demonstrate that ToF patients experience significant limitations, mainly on the physical aspects of HRQoL, a finding that is consistent with previous studies [16,17,18,19,20,21,22]. On the contrary, it seems from our results that ToF has no significant impact on mental health, a finding consistent with the results of a recent meta-analysis regarding mental health, where ToF patients did not report significant limitations in those domains nor in the summary of the mental component [6]. Adult patients with ToF reported poorer health in three out of four SF-36 scales, which contribute to the physical domain, meaning that they face limitations in their daily physical activity, or fulfilling their role at work, or experiencing more fatigue and poorer self-perceived health than their healthy counterparts. Interestingly, bodily pain scores did not differ significantly, even though all of the patients in the present study underwent surgery through median sternotomy, suggesting that pain is not a primary concern for ToF patients, but rather physical functionality is the key area of impact.

The substantial physical burden associated with the ToF condition on HRQoL was further derived by controlling for the age variable. Patients over 35 years of age performed poorly in the physical functioning scale, and this aligns with the midlife increase in adverse clinical events observed in patients with ToF and other types of congenital heart disease. While this may not be surprising given the general decline in health status with age, the assessment and understanding of aging-specific factors in the CHD population, such as frailty, sarcopenia, and biological markers of aging, has only recently become feasible due to improved survival into middle and older adulthood. Similar observations of poorer physical health status among older ToF patients have been reported by other researchers [23,24]. The time span from the operation could be another contributor to poor physical functioning since patients had been operated on in different time periods with the given surgical techniques. Loup et al. demonstrated that the more time that has passed from the last operation, the lower the scores in physical and mental health [25].

On the other hand, in our study, no extreme problems were reported to four dimensions (Mobility, Self-care, Usual Activities, and Pain/Discomfort), while some problems were most frequently reported in the EQ-5D dimensions of Anxiety/Depression and Pain/Discomfort, a common result in a previous study about self-assessed health status in adults with CHD [15] or in another general population study [16]. An interesting finding in the present study is that ToF patients reported a lower prevalence of problems in the Anxiety/Depression dimension of the EQ-5D compared to healthy controls. One possible explanation for this may lie in the structure of the EQ-5D instrument itself, which measures anxiety and depression in general terms using only three response levels. Additionally, it is worth noting that the control group was recruited during the COVID-19 pandemic, a period known to have a negative impact on mental health, which may have influenced their responses.

Of particular interest, reoperated ToF patients did not demonstrate a significant reduction in HRQoL compared to those without reintervention. While repeated surgical procedures might be anticipated to negatively influence physical or psychological well-being [21,23], our findings suggest that, with appropriate management and follow-up, reoperation does not necessarily lead to diminished perceived quality of life.

Another notable finding is that ToF patients reported higher Vitality scores compared to their healthy peers. This was particularly evident among individuals in the “perfect health” group (EQ-5D = 1), where both patients and controls reported no problems across any EQ-5D dimensions. It is important to highlight that all ToF patients in our cohort had undergone complete surgical repair with a transannular patch, potentially enabling them to lead relatively normal lives postoperatively. Previous research on the quality of life of patients with congenital heart disease has underscored the significant positive impact of corrective surgery on HRQoL, as such interventions are often curative and facilitate a return to normal life [26]. However, in the “any problems” group (EQ-5D < 1), the statistical significance of Vitality scores was lost, highlighting the influence of residual health issues or disease severity on HRQoL.

With regard to the associations between SF-36 and EQ-5D dimensions, an unexpected result emerged: the EQ-5D Mobility dimension did not correlate with the SF-36 Physical Functioning scale, nor did EQ-5D Usual Activities correlate with the SF-36 Role Physical scale. In contrast, the EQ-5D Pain/Discomfort dimension showed a moderate correlation with the SF-36 Bodily Pain scale. These discrepancies likely reflect the structural limitations of the EQ-5D, which employs fewer response options and less granularity than the more detailed SF-36. This suggests that, particularly for ToF patients, the use of only generic instruments may not adequately capture the full spectrum of HRQoL, and that incorporating disease-specific questionnaires alongside generic tools would provide a more comprehensive assessment.

Beyond these structural considerations, the significant results from both generic instruments (EQ-5D and SF-36) revealed an intriguing contrast. In domains assessing physical aspects of health, such as SF-36 Physical Functioning and EQ-5D Mobility, adult ToF patients reported worse health than their healthy peers. Conversely, when evaluating mental health-related dimensions, such as SF-36 Vitality and EQ-5D Anxiety/Depression, ToF patients appeared to report better outcomes than controls, particularly when stratified by health state. This pattern suggests that while ToF may have a lasting negative impact on physical functioning, patients may develop adaptive psychological coping mechanisms, resulting in better perceived mental and emotional well-being than their healthy counterparts.

This study has some limitations that should be taken into account. First, the relatively small patient sample size (which drags the sample size of healthy controls), despite the rarity of the disease. Secondly, the lack of data, such as socio-economic characteristics, comorbidities, health habits, etc., in order to study the factors that influence HRQoL. Thirdly, the fact that HRQoL was measured using two generic instruments, even though they have been used in general and disease-specific population studies. In case of rare diseases such as ToF, more information about health domains may be required in order to shed more light on the impact of ToF on HRQoL, and the need to use a separate disease-specific instrument, alongside the generic instruments, seems necessary.

## 6. Conclusions

In conclusion, this study assessed and compared the quality of life in adults after repair of ToF with that of healthy control peers. The self-perceived physical health was significantly poorer in ToF patients than in controls, whereas the results showed no statistical significance for mental health. As mentioned above, tetralogy of Fallot is a rare heart disease; however, on the other hand, improvements in medicine have raised the life expectancy of ToF patients. Nevertheless, future studies involving a large sample of patients, using disease-specific alongside the generic instruments, will permit researchers to have information on specific aspects of ToF patients’ HRQoL.

## Figures and Tables

**Table 1:** Demographic and health-related characteristics of the whole sample.

Variables	N	%
Sex		
Men	74	65.5
Women	39	34.5
Age		
18–24	30	26.5
25–34	44	38.9
35–44	17	15.0
45+	22	19.5
Patient status		
Patients	53	46.9
Controls	60	53.1
Reoperation		
Men	28	
Women	13	

**Table 2:** SF-36 scales and summary scores, EQ index, and EQ-5D VAS mean scores in total sample and patient–control subgroups according to sex and age.

	PF	RP	BP	GH	VT	SF	RE	MH	PCS	MCS	EQ-5D	EQ-VAS
**Total**												
**Patient status**												
Patients	85.2	74.1	85.9	68.9	66.8	80.9	79.2	68.7	51.3	48.2	0.81	80.09
Controls	93.4	89.6	84.0	77.4	64.8	84.2	82.8	71.5	54.6	48.4	0.89	86.92
Sig.	<0.001 *	<0.05 *	ns *	<0.05 **	ns *	ns *	ns *	ns *	<0.05 **	ns *	ns *	<0.0001 *
**Sex**												
Men	91.3	78.4	85.6	73.3	68.6	83.4	81.1	72.2	52.9	49.3	0.88	84.53
Women	86.3	89.7	83.5	71.7	60.4	81.1	81.2	66.2	53.3	46.5	0.80	82.18
Sig.	ns *	ns *	ns *	ns **	ns *	ns *	ns *	ns *	ns **	ns *	ns *	ns *
**Age**												
18–24	92.8	85.8	85.2	71.5	57.7	78.7	72.2	63.7	54.9	43.7	0.80	81.83
25–34	91.7	84.7	86.8	75.0	68.6	86.6	87.1	74.4	53.4	50.5	0.88	85.80
35–44	91.2	77.9	84.0	76.6	67.1	80.2	80.4	68.9	53.5	47.6	0.82	81.47
45+	79.5	76.1	81.4	70.2	70.0	81.8	81.8	71.4	49.6	50.4	0.87	83.86
Sig.	<0.05 *	ns *	ns *	ns **	ns *	ns *	ns *	ns *	ns **	ns *	ns **	ns **
**Patients**												
**Sex**												
Men	87.4	67.1	88.1	69.4	70.3	81.8	77.1	71.5	51.2	49.1	0.83	80.57
Women	80.8	87.5	81.8	67.9	60.0	79.2	83.3	63.1	51.6	46.4	0.76	79.17
Sig.	ns *	<0.05 *	ns *	ns **	ns *	ns *	ns *	ns *	ns **	ns *	ns *	ns *
**Age**												
18–24	88.5	76.5	83.2	65.6	56.5	79.4	76.5	62.4	52.2	44.5	0.76	78.53
25–34	87.8	79.7	86.2	69.6	71.9	84.4	85.4	74.3	51.6	51.0	0.85	81.25
35–44	86.7	63.9	87.5	73.5	63.3	77.8	74.1	68.4	51.6	46.8	0.75	75.56
45+	75.0	70.5	88.5	69.1	78.2	80.7	78.8	70.5	49.4	50.9	0.86	84.55
Sig.	<0.05 *	ns *	ns *	ns **	ns *	ns *	ns *	ns *	ns **	ns *	ns *	ns *
**Controls**												
**Sex**												
Men	94.7	88.5	83.5	78.8	67.1	84.9	84.6	72.8	54.4	49.4	0.92	88.10
Women	90.9	91.7	84.9	74.9	60.7	82.7	79.4	68.9	54.7	46.6	0.83	84.76
Sig.	ns *	ns *	ns *	ns **	ns *	ns *	ns *	ns *	ns **	ns *	ns *	ns *
**Age**												
18–24	98.5	98.1	87.8	79.9	59.2	77.9	66.7	65.5	58.8	42.5	0.85	86.15
25–34	93.9	87.5	87.1	78.1	66.8	87.9	88.1	74.4	54.4	50.2	0.90	88.39
35–44	96.2	93.7	80.0	80.0	71.2	82.8	87.5	69.5	55.5	48.5	0.89	88.13
45+	84.1	81.8	74.5	71.4	61.8	82.9	84.8	72.4	49.8	49.9	0.89	83.18
Sig.	<0.05 *	ns *	ns *	ns **	ns *	ns *	ns *	ns *	<0.05 **	ns *	ns *	ns *

PF = Physical Functioning, RP = Role Physical, BP = Bodily Pain, GH = General Health, VT = Vitality, SF = Social Functioning, RE = Role Emotional, MH = Mental Health. PCS = Physical Component Score, MCS = Mental Component Score. * Non-parametric Mann–Whitney test and Kruskal–Wallis test were performed. ** Parametric *t*-test and One-way ANOVA test were performed, ns: non-significant.

**Table 3:** Prevalence and severity of health problems using the EQ-5D-3L in the patient and control subgroups.

	Patients			Controls			
	No	Some	Extreme	No	Some	Extreme	Sig *
	Problem	Problem	Problem	Problem	Problem	Problem	
**MO**	46 (86.8)	7 (13.2)	-	60 (100)	-	-	<0.0001
**SC**	52 (98.1)	1 (1.9)	-	59 (98.3)	1 (1.7)	-	ns
**UA**	46 (86.8)	7 (13.2)	-	53 (88.3)	7 (11.7)	-	ns
**PD**	41 (77.4)	12 (22.6)	-	52 (86.7)	8 (13.3)	-	ns
**AD**	29 (57.7)	16 (30.2)	8 (15.1)	31 (51.7)	27 (45.0)	2 (3.3)	<0.05

MO = Mobility, SC = Self-care, UA = Usual Activities, PD = Pain/Discomfort, AD = Anxiety/Depression. * Chi square test, ns: non-significant.

**Table 4:** SF-36 mean scores using EQ-5D_index._

SF-36	Patients	EQ-5D = 1			EQ-5D < 1	
		Controls	Sig.	Patients	Controls	Sig.
		(*n* = 25)	(*n* = 30)		(*n* = 28)	(*n* = 30)
**PF**	92.60	97.66	<0.05 *	78.57	89.17	<0.05 *
**RP**	81.00	93.33	<0.05 *	76.86	85.83	<0.05 *
**BP**	94.12	89.36	ns *	78.61	78.63	ns *
**GH**	72.08	83.83	<0.05 **	66.11	71.30	ns **
**VT**	80.60	72.66	<0.05 *	54.46	57.00	ns *
**SF**	93.50	90.00	ns *	69.64	78.33	ns *
**RE**	89.33	94.44	ns *	70.33	71.11	ns *
**MH**	80.16	81.20	ns *	58.43	61.73	ns *
**PCS**	53.08	55.40	<0.05 **	49.78	53.81	ns **
**MCS**	54.31	53.65	ns *	42.70	43.28	ns *

PF = Physical Functioning, RP = Role Physical, BP = Bodily Pain, GH = General Health, VT = Vitality, SF = Social Functioning, RE = Role Emotional, MH = Mental Health. PCS = Physical Component Score, MCS = Mental Component Score. * Mann–Whitney test, ** *t*-test, ns: non-significant.

**Table 5:** Spearman correlations between the SF-36 and EQ-5D scales, EQ-5D index, and EQ-VAS in the patient group.

	PF	RP	BP	GH	VT	SF	RE	MH	PCS	MCS
**MO**	−0.14	−0.20	−0.06	−0.07	−0.26	−0.26	−0.13	−0.31 *	−0.04	−0.28 *
**SC**	−0.16	0.06	−0.12	−0.13	−0.10	−0.14	−0.10	−0.12	−0.14	−0.05
**UA**	−0.01	0.02	−0.07	−0.03	−0.34 *	−0.11	−0.26	−0.47 **	−0.13	−0.46 **
**PD**	−0.47 **	−0.11	−0.58 **	−0.28 *	−0.54 **	−0.18	−0.03	−0.47 **	−0.28 *	−0.45 **
**AD**	−0.34 *	−0.23	−0.36 *	−0.16	−0.50 **	−0.50 **	−0.19	−0.41 **	−0.25	−0.53 **
**EQ-5D_index_**	0.37 **	0.15	0.37 **	0.11	0.62 **	0.46 **	0.20	0.60 **	0.15	0.65 **
**EQ-VAS**	0.30 *	0.05	0.26	0.27 *	0.44 **	0.31 *	0.26	0.31 *	0.14	0.49 **

PF = Physical Functioning, RP = Role Physical, BP = Bodily Pain, GH = General Health, VT = Vitality, SF = Social Functioning, RE = Role Emotional, MH = Mental Health. PCS = Physical Component Score, MCS = Mental Component Score. MO = Mobility, SC = Self-care, UA = Usual Activities, PD = Pain/Discomfort, AD = Anxiety/Depression. * Significant at the 0.05 level. ** Significant at the 0.01 level.

## Data Availability

The original contributions presented in this study are included in the article. Further inquiries can be directed to the corresponding author.
